# Climate-modulated upwelling drives phytoplankton variability and biomass connectivity in the Humboldt Archipelago coastal system

**DOI:** 10.1038/s41598-026-56707-y

**Published:** 2026-06-11

**Authors:** Victor M. Aguilera, Matthew L. Hammond, Iván Pérez, Pablo Gorostiaga

**Affiliations:** 1 Centro de Estudios Avanzados en Zonas Áridas (CEAZA), Coquimbo, Chile; 2 Facultad de Ciencias del Mar, Depto. Biología Marina, Universidad Católica del Norte, Coquimbo, Chile; 3 Centro i-mar, Universidad de Los Lagos, Puerto Montt, Chile; 4 Centro de Investigación Oceanográfica COPAS COASTAL, Universidad de Concepción, Concepción, Chile

**Keywords:** Climate sciences, Ecology, Ecology, Ocean sciences

## Abstract

Marine productivity driven by phytoplankton biomass (chlorophyll-a, Chl) sustains biodiversity, fisheries, and ecosystem services in the Humboldt Archipelago (29°S), an arid coastal upwelling region within the Humboldt Current System. The archipelago is characterized by strong bathymetric gradients and a submarine canyon that generate complex circulation patterns, potentially affecting phytoplankton aggregation. Although upwelling occurs year-round, its efficiency varies seasonally and interannually under the influence of regional atmospheric forcing and large-scale climate modes, including the El Niño–Southern Oscillation (ENSO) and the Pacific Meridional Mode (PMM). We evaluated how local hydrographic structure, upwelling dynamics, and climate variability interact to regulate Chl variability and biomass connectivity within the Coquimbo–Humboldt upwelling system. To address this, CTD-fluorescence profiles collected at a fixed site during 21 near-monthly surveys between November 2022 and December 2024 were combined with satellite observations and atmospheric data. Chlorophyll variability was analyzed using hierarchical Generalized Additive Models, of which the baseline physical model explained 80% of Chl deviance, while inclusion of the PMM increased it to ~ 84% and substantially improved model performance. Seasonal Chl variability was primarily associated with mixed-layer depth shoaling and intermediate Ekman transport, indicating strong control by short-term physical forcing. In contrast, the PMM-Ekman transport interaction revealed that upwelling efficiency depended on the large-scale climatic background. Satellite observations further suggested the episodic export of phytoplankton biomass from the Coquimbo Bay system into the archipelago. These results demonstrate that climate modes modulate local upwelling efficiency, shaping phytoplankton dynamics in one of the most biodiverse coastal regions of the Southeastern Pacific.

**Supplementary Information:**

The online version contains supplementary material available at 10.1038/s41598-026-56707-y.

## Introduction

Marine productivity and related ecosystem services, such as climate regulation, food provision, and biodiversity conservation, are mediated by the temporal and spatial dynamics of phytoplankton biomass^1^. Phytoplankton, unicellular photoautotrophic microorganisms that form the basis of the ocean’s food web^1^, contribute to half of global net primary production by chemically reducing atmospheric CO_2_ to produce organic molecules^2^. Productivity, by definition, is the biomass-specific growth rate of a population or a functional phytoplankton group. Phytoplankton biomass can be estimated through fluorescence measurements as a proxy for chlorophyll-a (Chl) concentration^3^. Fluorometers installed on CTDs (conductivity, temperature, and depth) and radiometers on satellites allow the non-invasive assessment of variations in Chl over a wide range of sampling scales (from hours to decades), which are evident both horizontally and vertically^2,4^. Nonetheless, CTD fluorescence profiles might underestimate Chl in surface waters during daytime (especially at noon), due to fluorescence quenching^5,6^. Meanwhile, satellite-based methods are limited to cloud-free conditions^7^ and detect not only fluorescence but also other light-reactive substances^8^. Thus, it is necessary to properly address and validate vertical profiles of fluorescence, as well as the resolution and coverage of satellite images, with the aim of accurately retrieving spatial-temporal variations in Chl concentration^9^.

Phytoplankton biomass is higher near continental edges, particularly in Eastern Boundary Upwelling Systems (EBUS)^10^. In these regions, wind-driven offshore transport promotes the upwelling of cold, nutrient-rich subsurface water onto the continental shelves, fostering elevated levels of Chl^11^. Superimposed on this large-scale spatial pattern, a suite of mesoscale and sub-mesoscale processes such as eddies, fronts, and filaments strongly influences the transport, mixing and retention of phytoplankton biomass^12,13^. Coastal topography, including bathymetry and shoreline configuration, can further modulate these processes by generating localized retention areas and circulation features. Vertically, the seasonal dynamics of the mixed layer depth (MLD) also contribute to changes in phytoplankton biomass^14^. Linked, yet not linearly, to wind intensity^10^, the MLD regulates availability and mode of supply of nutrients to a specific environment, and light availability^14^. In turn, stratification of the water column might determine the source (and nutrient load) of the upwelled water, with deeper upwelling occurring in less stratified waters^15,16^.

**Fig. 1:**
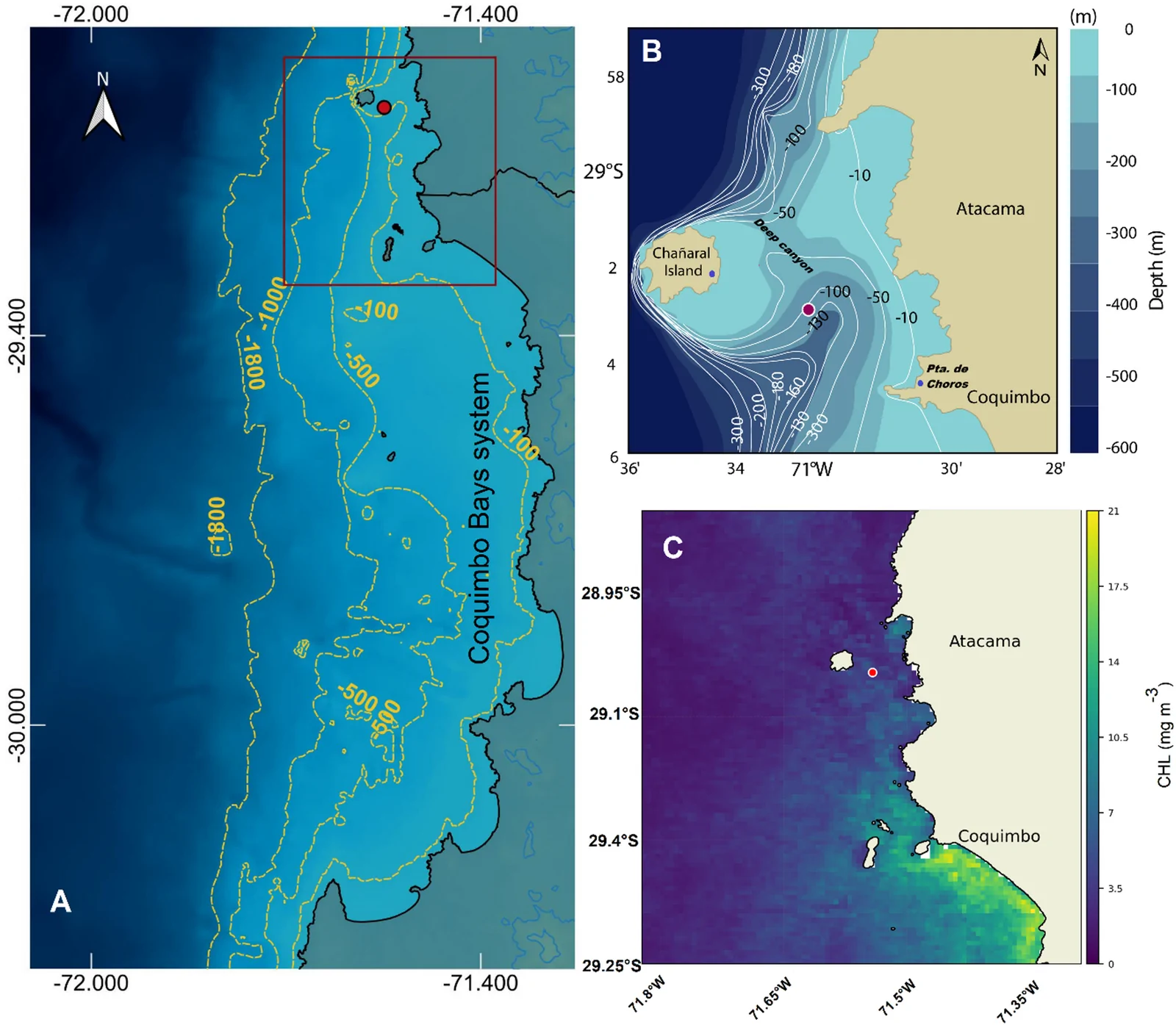
(A) Study area illustrating the Coquimbo Bay system and the Humboldt Archipelago to the north (red square). B) Magnification of the study area highlighting the locations of weather stations on Chañaral Island and at Punta de Choros (blue dots), and in situ hydrographic measurements above the deep canyon crossing the archipelago (red dot). C) Spatially broader composite of satellite CHL distribution.

The Coquimbo upwelling system constitutes an important subtropical (29–30 °S) and arid upwelling region within the Humboldt Current System (HCS) (Fig. 1), in the Southeastern Pacific^17^. It harbors important geographic milestones, including headlands, aquaculture and recreational bays and the Humboldt Archipelago. Housing two marine reserves that provide habitat for a highly diverse and endemic marine fauna, the Humboldt Archipelago (HA) is regarded as a global biodiversity hotspot^18^. The HA is affected year-round by upwelling favorable winds, which increase significantly during the austral spring^19^. Upwelling filaments originating in southern neighboring upwelling centers^20^ connect the Coquimbo Bay system to the HA^21,22^. At the archipelago, currents and filaments containing a variable proportion of Sub-Antarctic Water (SAAW) and Equatorial Subsurface Water (ESSW)^23^ interact with a sharp zonal bathymetric gradient due to a deep canyon crossing the archipelago (Fig. 1B). This results in a complex sub-to-mesoscale circulation pattern^18,24^, modulated by intraseasonal meridional winds^21^. Given the upwelling-driven oceanic setting and the annual cycle of sunlight and cloud cover^25^, seasonal changes in phytoplankton biomass are expected in the HA. However, the regional atmospheric-oceanographic setting in the Coquimbo upwelling system can be substantially affected by the interaction between the El Niño Southern Oscillation (ENSO) and the Pacific Meridional Mode (PMM)^26–28^. During downwelling Kelvin waves, the warm El Niño phase deepens the thermocline, reducing upwelling efficiency, whereas the cold La Niña phase favors the reinforcement of upwelling, kinetic energy, and offshore transport in the northern HCS^29^. Meanwhile, the PMM develops in subtropical regions as southeast trade wind variability leading to a wind-evaporation-sea surface temperature (SST) equatorward propagation (WES feedback), surface wind anomalies, and latent heat flux anomalies^26–28^. In the southern hemisphere, the PMM is triggered by internal atmospheric variability associated with the South Pacific Oscillation during Austral winter^27,28^, manifesting in positive (warm)/negative (cold) phases consisting of SST anomalies extending southwestward from the subtropic region^26–28^. The alignment of warm/cold phases of the PMM and ENSO might amplify or depress positive or negative SST anomalies, respectively, in the equatorial Pacific and upwelling efficiency towards higher latitudes^27,30^. ENSO- and PMM-related oceanographic changes can modify the size-structure and abundance of phytoplankton in upwelling regions of the Humboldt and California EBUS^31,32^.

Despite its notable and highly endemic biodiversity and the associated human well-being, primarily evident through fishing and tourism, there is a significant lack of understanding of fundamental bio-oceanographic processes in the HA. In particular, the temporal and spatial dynamics of phytoplankton biomass, which largely determine the fitness of upper trophic populations inhabiting the archipelago, remain unstudied^24,33^. Such information is essential not only to explain and manage its highly endemic diversity under climate change, but also to address anthropogenic pressure^18^. Thus, this study aims to expand our understanding of HA functioning by examining the seasonal-to-interannual and spatial dynamics of phytoplankton biomass in relation to oceanic variables. We hypothesize that phytoplankton biomass in the HA is more sensitive to physical-chemical environmental perturbations associated with ENSO than to local/regional determinants. This was tested by analyzing CTD-fluorescence profiles obtained during 21 nearly monthly campaigns at a fixed site within the archipelago, from November 2022 to December 2024, encompassing different ENSO and PMM phases (Table 1). Satellite estimates of Chl, including spatially broader data (Fig. 1C), were used to test the spatial scope and complement in situ records. Field fluorescence data were tested for fluorescence quenching and converted to Chl. Spatiotemporal Chl variations were subsequently diagnosed through Generalized Additive Models (GAMs) to assess their relationship to hydrographic variability assessed as wind stress and oceanic processes (stratification, Ekman transport, Ekman layer, solar radiation), while accounting for seasonal, ENSO, and PMM phase variations.

**Table 1:** Date and sampling details of monthly surveys conducted over different seasons and ENSO phases.

Sampling Date	Austral season	ENSO phase	Discrete Chl-sampling	Day-night sampling	Offset value	Slope
2022-08-03	Winter	LN	-	-	0.110	0.64
2022-09-02	Spring	LN	-	-	0.082	2.50
2022-11-25	Spring	LN	Yes	-	0.110*	0.68
2022-12-16	Summer	LN	-	-	0.082	0.66
2023-04-02	Autumn	LN	Yes	-	0.093*	0.83
2023-05-10	Autumn	EN	Yes	-	0.093	0.64
2023-06-20	Autumn	EN	Yes	-	0.093	0.67
2023-07-14	Winter	EN	Yes	-	0.114	0.66
2023-10-13	Spring	EN	Yes	Yes	0.114	0.67
2023-11-30	Spring	EN	Yes	-	0.114	2.90
2024-01-09	Summer	EN	Yes	-	0.082*	2.50
2024-02-05	Summer	EN	Yes	Yes	0.114	1.23
2024-03-07	Summer	EN	Yes	Yes	0.114*	1.36
2024-04-02	Autumn	EN	Yes	-	0.093*	0.90
2024-05-12	Autumn	EN	-	Yes	0.082	2.50
2024-06-21	Winter	NEN	-	Yes	0.114	1.36
2024-07-14	Winter	NEN	-	Yes	0.114	0.66
2024-08-29	Winter	NEN	-	Yes	0.093	0.90
2024-10-06	Spring	NEN	-	-	0.093	0.90
2024-11-08	Spring	NEN	-	-	0.093	0.64
2024-12-04	Spring	NEN	-	-	0.114	2.90

Discrete, daytime, and nighttime sampling indicate the dates for fluorometric chlorophyll measurements and 24 h day-night sampling cycles. Offset values were measured in only a subset of surveys (*) and seasonally extrapolated. Slope denotes the correlation slope between fluorometric chlorophyll measurements and offset-corrected fluorescence profiles.

## Results

### Climate modes and water column response

**Fig. 2:**
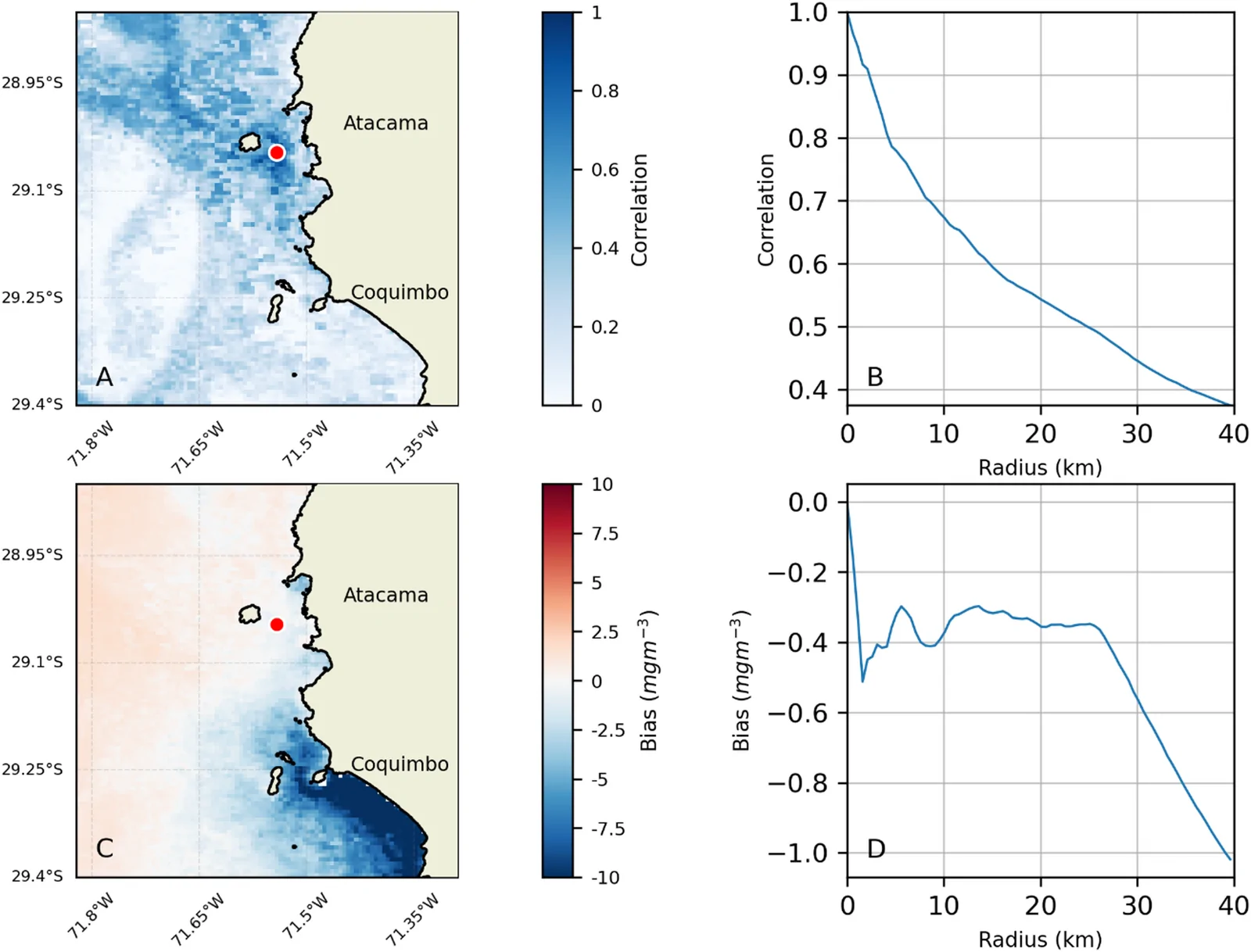
Remote, regional, and local oceanic conditions during the study period. (A) ENSO phases, (B) PMM activity, and in situ temperature (C), salinity (D), density (E), stratification (F), and dissolved oxygen (G).

The temporal evolution of the climate modes shows a positive SST anomaly of approximately 2° C in December 2023, but the warm phase extended for 16 months, from March 2023 to June 2024, highlighting the presence of an El Niño event (Fig. 2A). On the other hand, the PMM presented two warm phases, one from November 2022 to June 2023, showing the highest values of the index (1° C), and a second from January to May 2024 with a maximum PMM index of 0.5° C (Fig. 2B).

The water column structure reflected the influence of the annual cycle, showing the highest temperatures (≥ 17 °C), lower salinity (< 34.6 g kg^− 1^), lower density (25.75 kg m^− 3^), and oxygenated waters (≥ 250 µmol kg^− 1^) during the summers of 2023 and 2024 (Fig. 2C, D, E, G). This condition extended to approximately 20 m and contributed to water column stratification (Fig. 2F). Moreover, a deepening of the pycnocline was observed in two periods (e.g., autumn 2023 and summer 2024), favoring water column mixing and ventilation (Fig. 2E–G).

### Satellite images

**Fig. 3:**
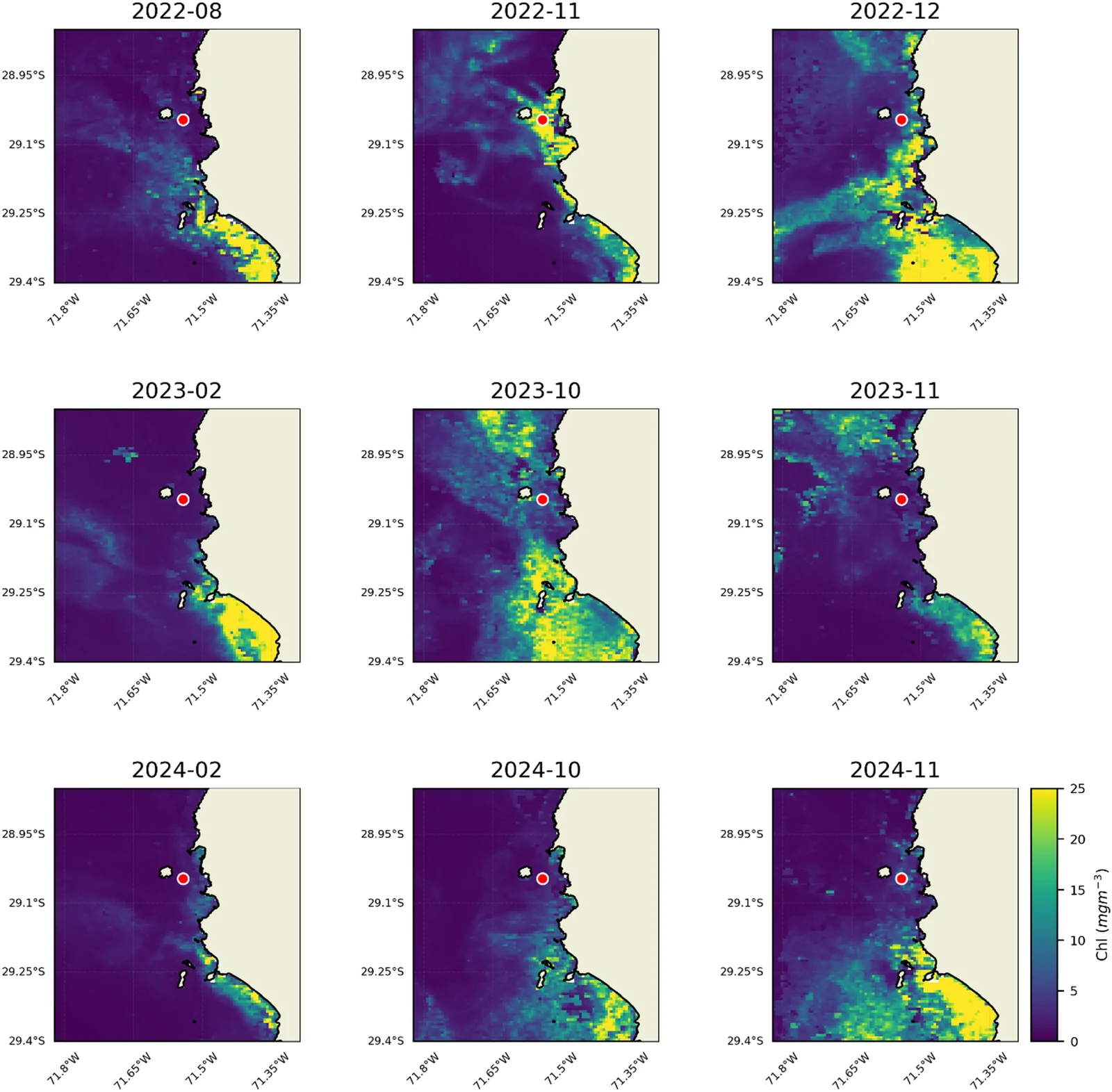
Spatial distribution of the CHL – Chl correlation (A) and its corresponding relationship with distance from the in situ sampling site (B). Spatial distribution of the bias of the CHL – Chl correlation (C) and its corresponding relationship with distance from the in situ sampling site (D).

**Fig. 4:**
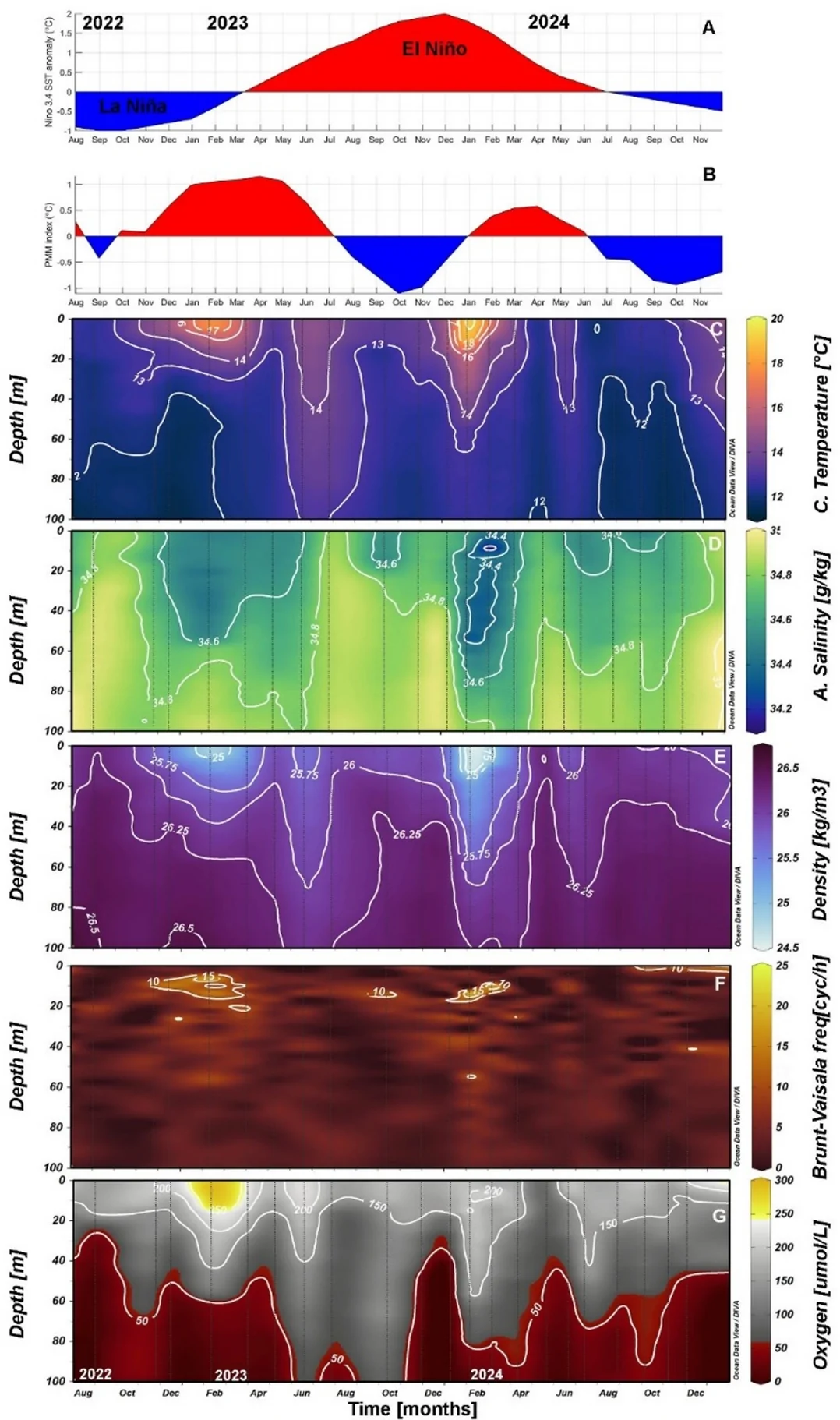
Spatially broader remote sensing images illustrating the magnitude and spatial coverage of phytoplankton blooms observed in the study area. The date of each image is included as their title. Note the massive CHL concentrations extending from the southern Coquimbo Bay system.

The spatial representativeness of the sampling site was assessed using remotely sensed surface chlorophyll (CHL) and its correlation and bias with in situ Chl records using two metrics: the correlation (i.e., the Pearson product-moment correlation coefficient) between the two time series and the bias (i.e., the average difference between the two time series) (Fig. 3). The correlation was generally strongest near the sampling point and towards the north up to 400–500 m (Figs. 3A-B), whereas it decreased towards Chañaral Island (Fig. 3C). The bias is divided into approximately three subregions: the north and west, where the sampling site overestimates by ~ 1 mg m^− 3^; the south and east, where the sampling site underestimates by ~6 mg m^− 3^; and an additional core close to the coast to the southeast with an even stronger negative bias (~-9 mg m^− 3^), likely due to the presence of strong Chl blooms in this subregion (Fig. 3D). Spatially broader satellite images during periods of elevated CHL concentrations (> 5 mg m^− 3^) suggest that phytoplankton blooms might originate from the southern Coquimbo Bay system (Fig. 4).

### Fluorescence to Chl conversion

**Fig. 5:**
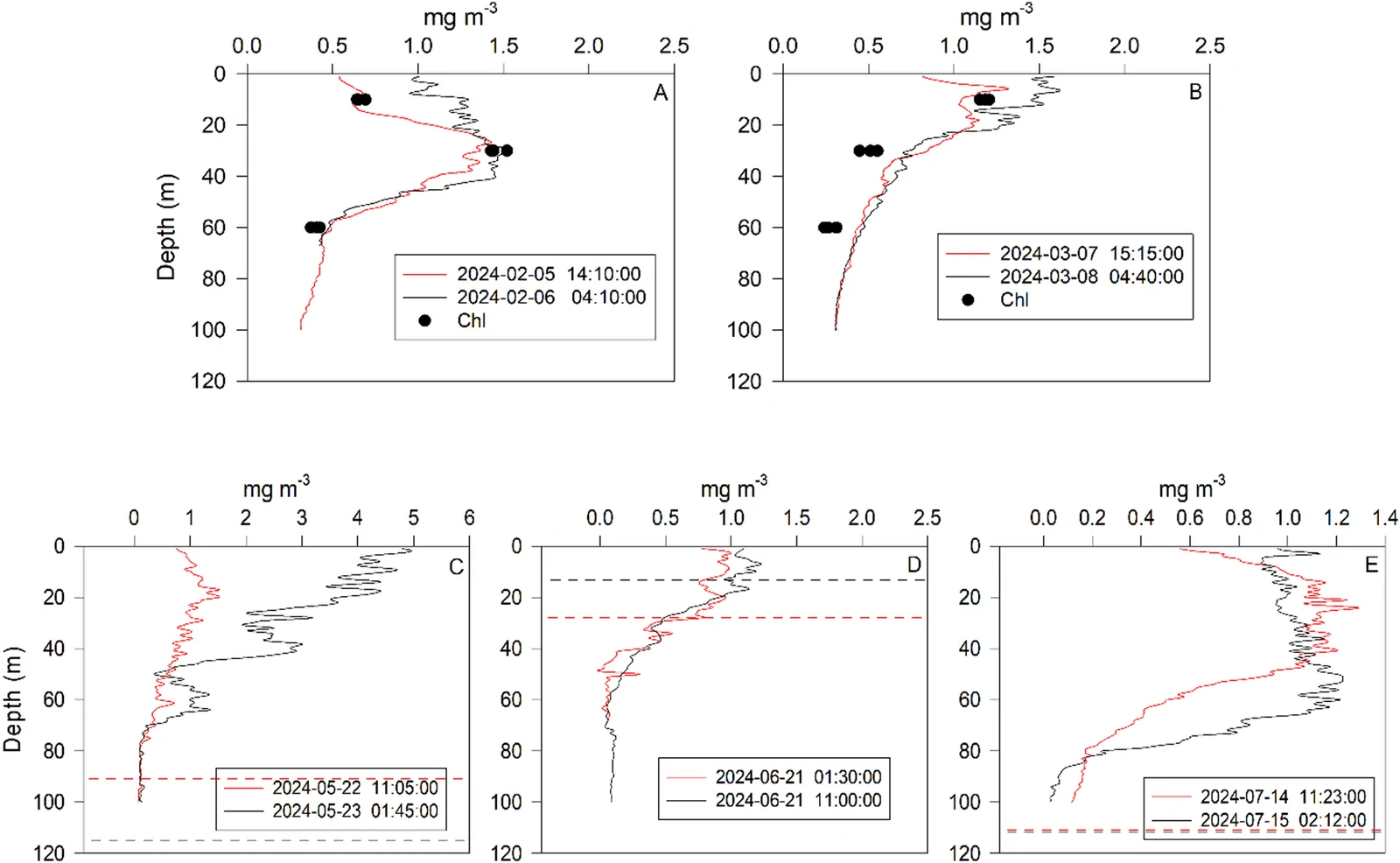
Photo-inhibition test and vertical distribution of day and night fluorescence profiles relative to the MLD. Day and night profiles and MLD are shown in red and black solid and dashed lines, respectively. A and B denote variable photoinhibition and fluorescence accuracy relative to discrete Chl measurements (black circles). C, D and E show the distribution of maximum fluorescence with regard to the MLD. Note that inhibition also occurs during autumn-winter months.

**Fig. 6:**
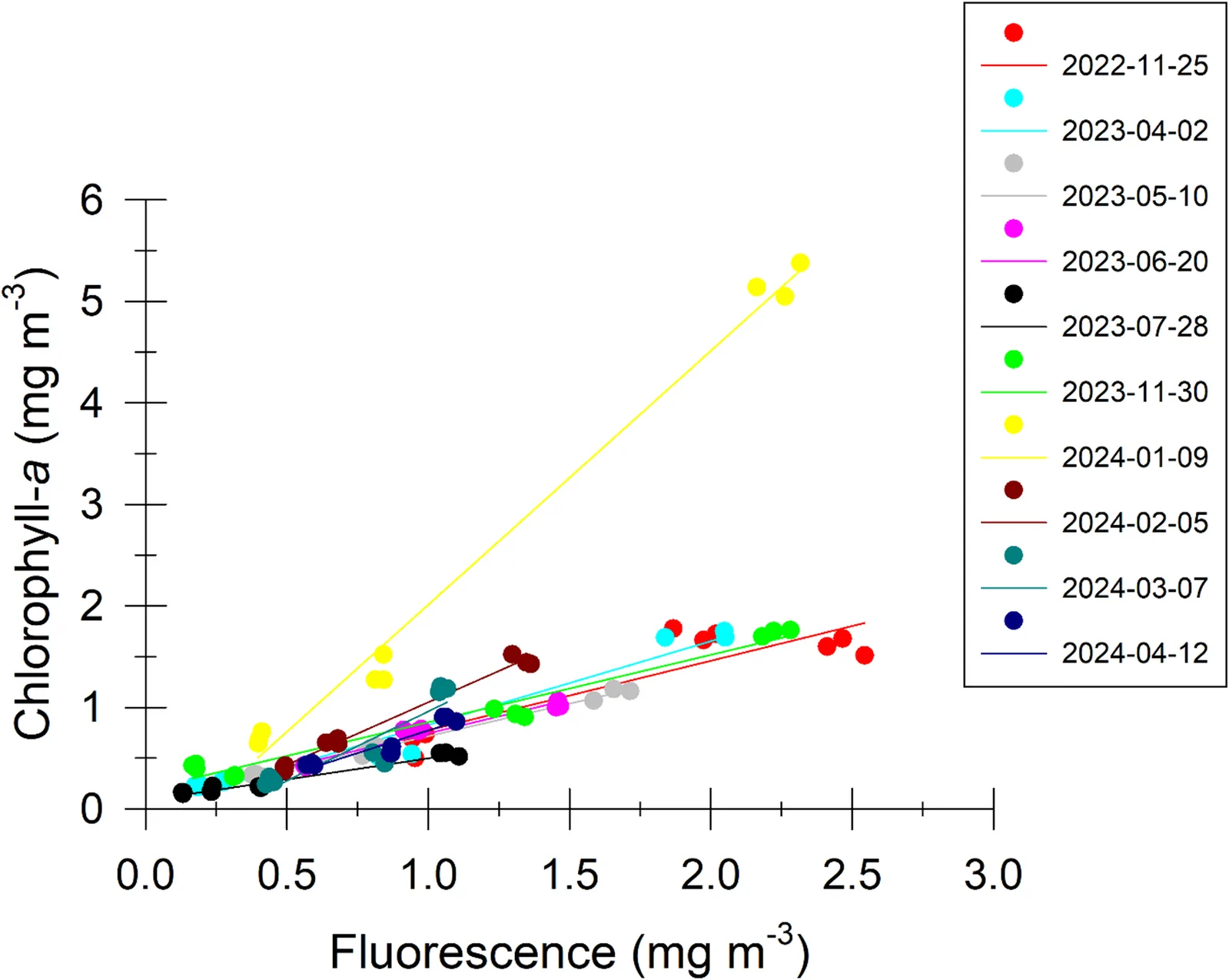
Linear correlations between offset-corrected and quenching-corrected fluorescence profiles and discrete Chl measurements for the different surveys. The slopes of these correlations facilitated the conversion of fluorescence into Chl profiles.

The comparison of day and night fluorescence profiles, including discrete Chl measurements and the location of the maximum fluorescence (Max_Fluo_) relative to the MLD, is shown in Fig. 5. This analysis revealed persistent photo-inhibition during daytime (Fig. 5A) and a subtle overestimation of Chl in fluorescence profiles relative to fluorometric measurements (Fig. 5B). Under a deep MLD, night fluorescence profiles displayed a quasi-uniform distribution at the surface, whereas a clear fluorescence depression appeared in day profiles because of fluorescence quenching (Fig. 5C). The Max_Fluo_ tends to define the deep quenching threshold, and thus, the depth of Max_Fluo_ is also considered in the fluorescence quenching correction (Table 2). The fluorescence quenching is evident even on occasions when the MLD is shallower (< 40 m depth) (Fig. 5D), whereas it tends to be less pronounced when the MLD is deep (> 80 m depth) and Max_Fluo_ is in the subsurface (Fig. 5E). The statistical results of the distribution of the day-night fluorescence ratio in the mixed layer (upper and lower) are shown in Sup. Mat. (Fig. S1). Despite the occurrence of fluorescence quenching in the upper layer, the analysis did not detect differences between the upper and lower layers of the mixed layer. Both upper and lower layers showed day-night fluorescence ratio values < 1. Nevertheless, higher values were mostly found in the lower layer. Significant correlations with variable slopes were found between fluorometric Chl measurements and the offset, and between fluorescence quenching-corrected fluorescence profiles and the offset for the different surveys (Fig. 6).

**Table 2:** Summary of day-night sampling conducted to evaluate the quenching effect.

Sampling	MLD		Max_Fluo_		Z_MaxFluo_	
	Day	Night	Day	Night	Day	Night
Oct-23	14	15	1.29	1.64	3	16
Feb-24	12	9	1.35	1.54	34	30
Mar-24	9	12	1.31	1.58	6	1
May-24	93	124	1.52	4.96	20	2
Jun-24	28	17	0.99	1.23	3	7
Jul-24	110	110	1.29	1.23	24	51
Aug-24	44	33	2.24	0.9	13	10

Variables assessed included mixed layer depth (MLD), maximum fluorescence (Max_Fluo_) and depth of Max_Fluo_ (Z_MaxFluo_).

### Temporal and vertical variations in Chl

**Fig. 7:**
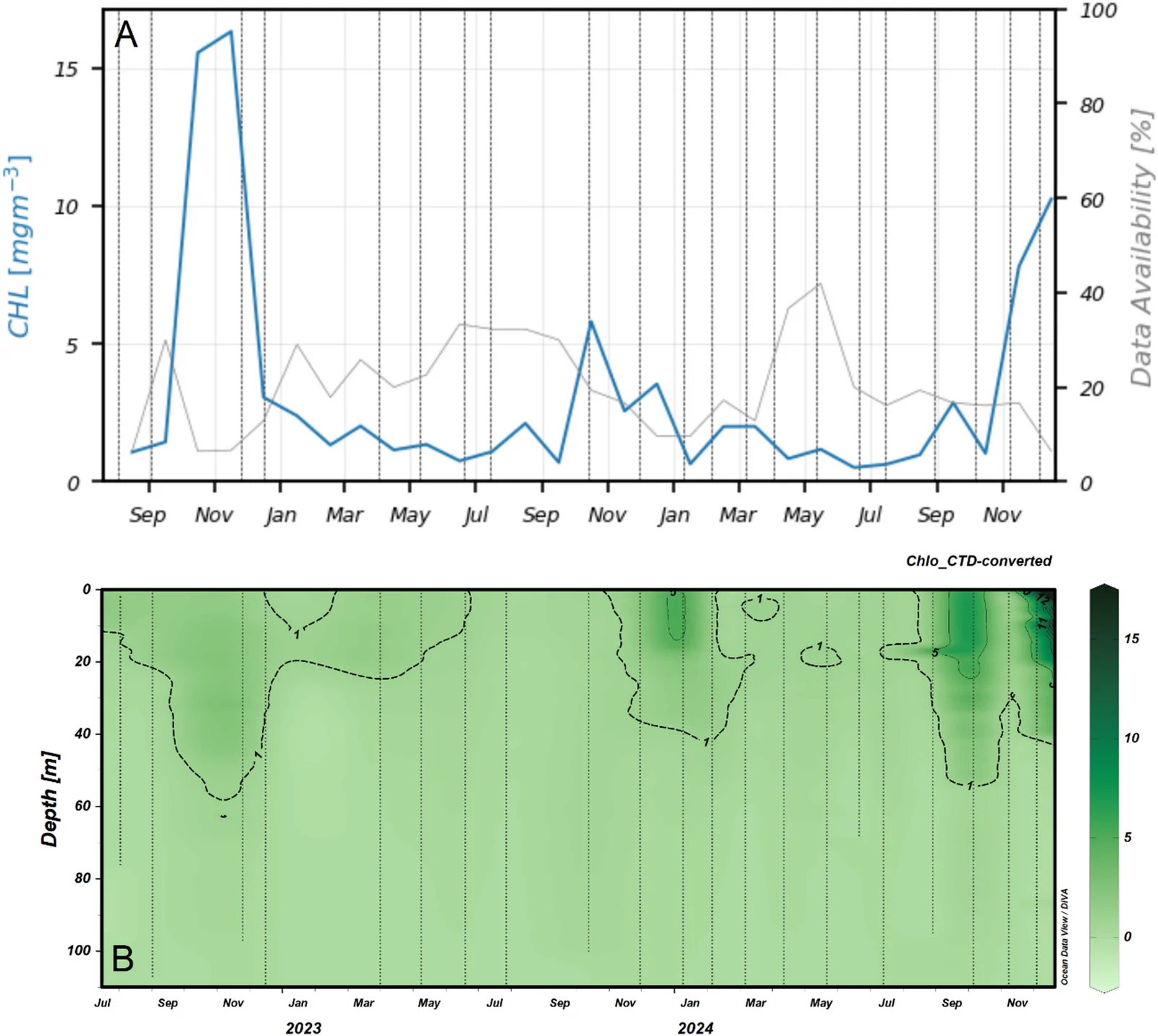
Time series of remotely sensed CHL (A) and in situ Chl measurements with the corresponding percentage of days in each month with data (B). Note that the CHL maximum observed during November 2022 was not captured by in situ monitoring.

The temporal and vertical variations in phytoplankton biomass over the 2022–2024 period are illustrated through the satellite CHL time series and vertical in situ Chl sections (Fig. 7). Subtly overestimating in situ Chl concentrations, the CHL time series revealed peaks (> 5 mg m^− 3^) in the concentration during spring months (October 2022, October 2023, and December 2024) (Fig. 7A). These peaks were relatively higher (> 10 mg m^− 3^) during 2022 and 2024. Except for the CHL peak of October 2022, in situ campaigns captured most of the temporal Chl variations in the study section as depicted through the satellite CHL time series (Fig. 7B). The results indicate that the HA corresponds to a mesotrophic ecosystem, where low to moderate Chl concentrations (1–2 mg m^− 3^) prevail throughout the year, with a significant increase in spring. Vertically, Chl tends to be concentrated in the upper 10–30 m, but in spring, the MLD can reach 40–60 m (Fig. 7B).

**Fig. 8:**
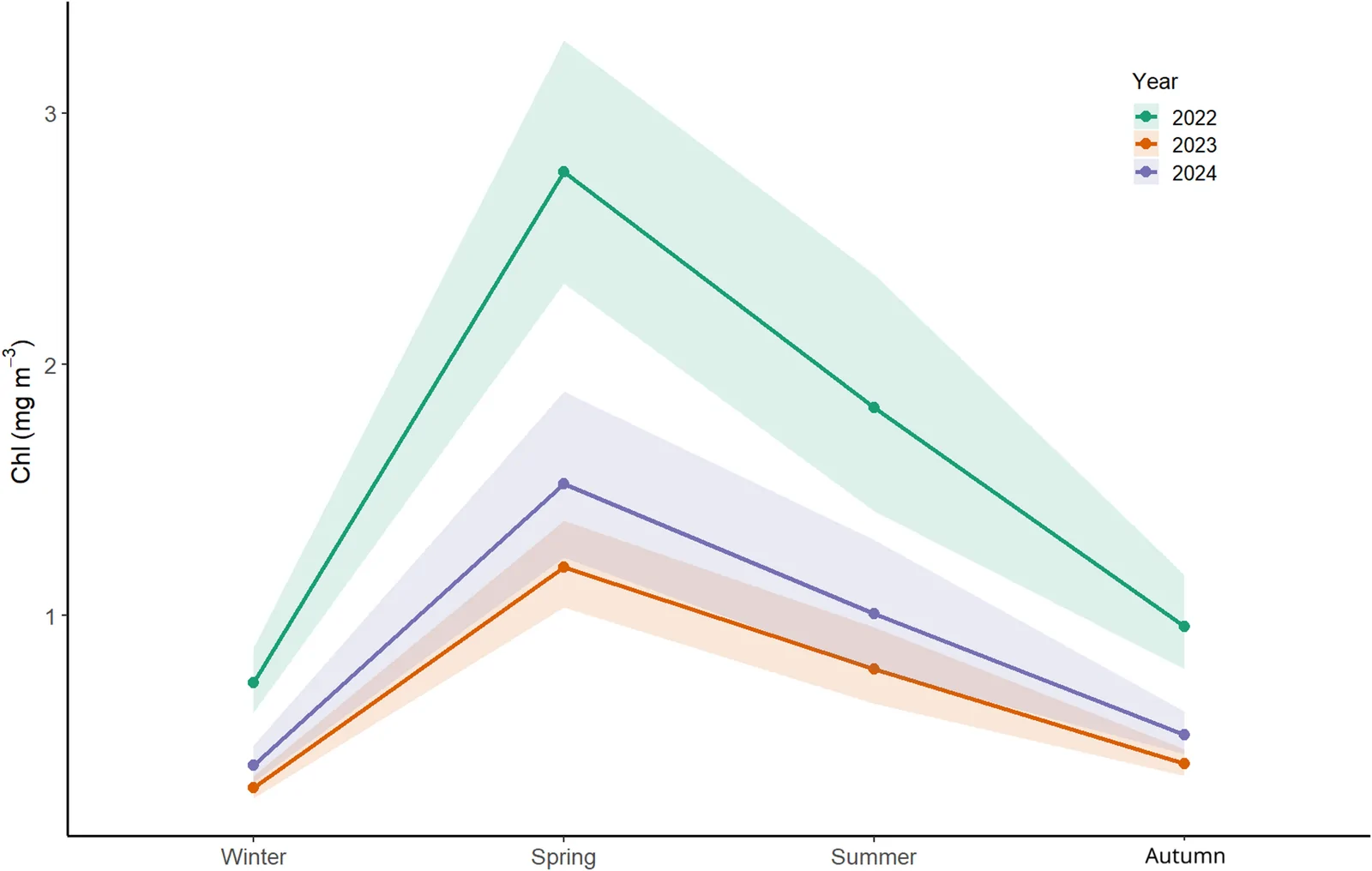
Seasonal and interannual Chl means (± 95% IC) observed in the study area. GAM-adjusted predictions were generated for all possible combinations of season and year during the construction of the prediction grid.

**Fig. 9:**
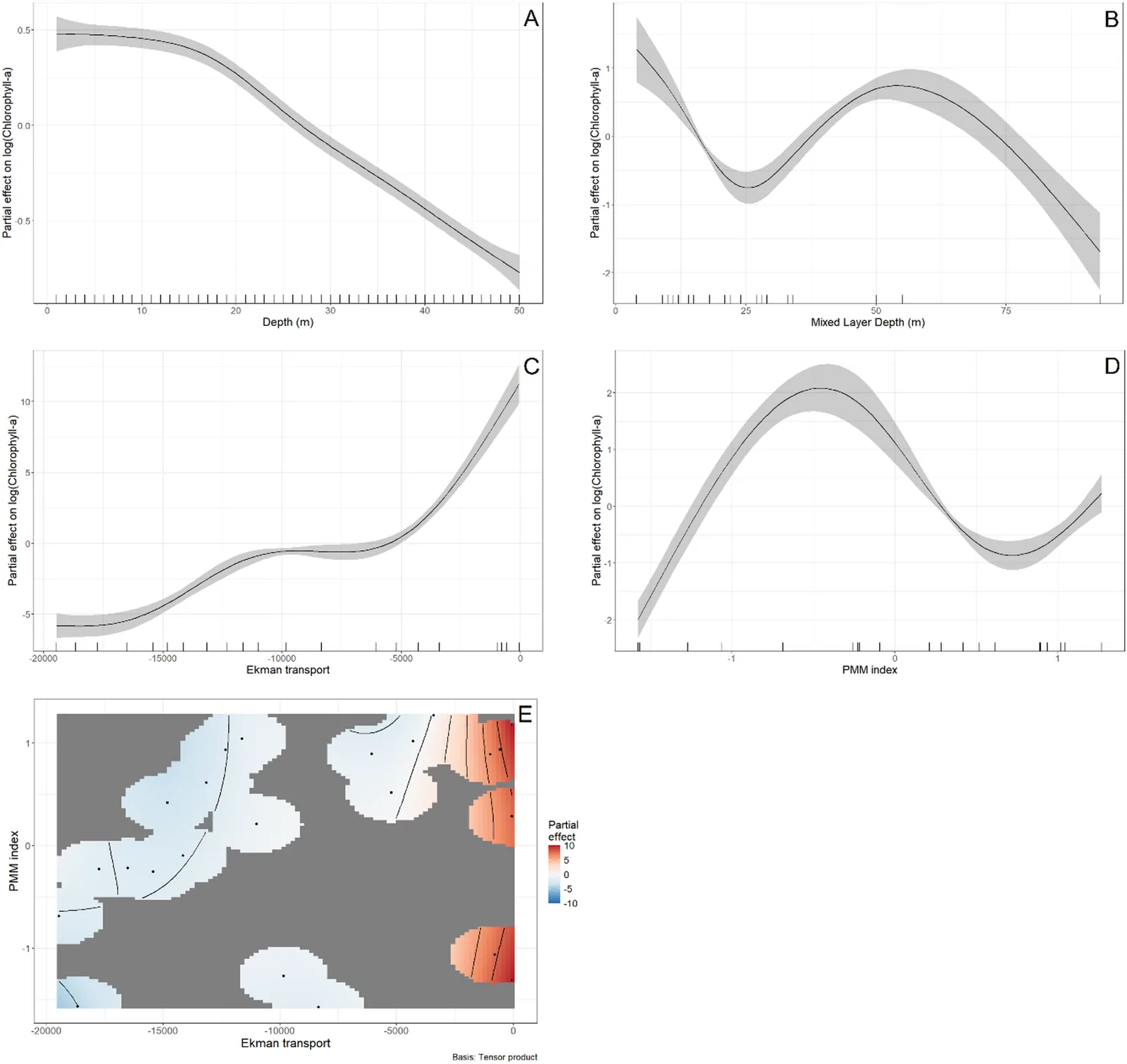
Smooth effect of main oceanographic drivers of Chl temporal and vertical variations. (A) Depth, (B) MLD, (C) Ekman transport, (D) PMM and tensor product Ekman*PMM.

The baseline physical Model A (Tables 3 and 4) explained a substantial proportion (≈ 80% of the deviance) of in situ Chl variability, confirming that short-term physical processes dominate phytoplankton’s biological response. On a seasonal scale, Chl concentration was significantly higher during the spring and summer seasons (Fig. 8). The Chl concentration observed during 2023 was significantly lower than that of 2022 and 2024. The influence of physical variables (smooth terms) was highly significant, indicating strong non-linear relationships between Chl concentration and vertical structure as well as recent upwelling intensity (Fig. 9). The Chl concentration reached maximum values below the surface at intermediate depths and declined rapidly below this layer (Fig. 9A). The smooth term for MLD showed that Chl was highest at relatively shallow MLD and decreased as MLD became deeper, with a marked decline beyond intermediate depths (Fig. 9B). The Chl concentration increased with Ekman transport up to an intermediate range, beyond which the effect weakened or plateaued (Fig. 9C). These findings indicate that moderate upwelling-favorable winds enhance phytoplankton biomass, whereas stronger upwelling and Ekman transport do not. The smooth effect of PMM indicated maximum Chl concentrations at moderately negative PMM values ( ~ − 0.5) rather than during extreme PMM phases (Fig. 9D).

**Table 3:** escription of hierarchical GAMs applied to elucidate the roles of the local concurrent state and regional ocean background in controlling the temporal and vertical dynamics of Chl in the Humboldt Archipelago.

Model	Formula	Description
A	Chl ~ Season + Year + s(Depth, k = 10) + s (MLD, k = 5) + s(EkmanAcu, k = 5)	Phytoplankton response to immediate physical state
B	Chl ~ Season + Year + s(Depth, k = 10) + s (MLD, k = 6) + s(EkmanAcu, k = 6) + s(PMM, k = 5)	Phytoplankton response to immediate physical state and its low-frequency climatic modulator
C	Chl ~ Season + Year + s(Depth, k = 10) + s (MLD, k = 6) + te(EkmanAcu, PMM, k = c(5, 5))	Interaction between local upwelling dynamics and climatic state through a tensor product

**Table 4:** Summary of parametric coefficients from the statistical evaluation of temporal Chl variations.

Term	Estimate	Std. Error	t value	Pr(>|t|)
(Intercept)	-0.31699	0.04143	-7.652	4.59e-14 ***
Season*Spring	1.04304	0.04505	23.154	< 2e-16 ***
Season*Summer	1.05640	0.07114	14.849	< 2e-16 ***
Season*Autumn	-0.11143	0.05954	-1.872	0.0616
Year 2023	-0.70845	0.04768	-14.860	< 2e-16 ***
Year 2024	-0.08785	0.04948	-1.775	0.0761

Seasonal and interannual comparisons were made relative to winter and the year 2022, respectively. Pr(>|t|) is the p-value of t-statistic. Significant codes: 0 ‘***’ 0.001 ‘**’ 0.01 ‘*’ 0.05 ‘.’ 0.1 ‘ ’ 1.

Model B, which incorporated the PMM as a low-frequency climatic modulator, led to a marked improvement in model performance (Table 5). According to the Akaike Information Criterion (AIC), which sizes the trade-off between log-likelihood and model complexity, the inclusion of the PMM led to a large decrease in the AIC (-225) and an increase in explained deviance (84.2%). Furthermore, the PMM accounted for additional interannual variability not captured by local physical drivers or ENSO alone. The PMM smooth term was highly significant, while the physical predictors of Model A retained their explanatory power. This confirms that the PMM effect (climatic PMM variability) operates in addition to, rather than instead of, local forcing. Finally, Model C (interaction between local upwelling dynamics and climatic state) yielded the lowest AIC and comparable explained deviance (84.3%) to Model B, indicating that the biological response to upwelling is conditional on the prevailing climatic background. The significant Ekman × PMM interaction indicates that similar upwelling intensities can yield markedly different Chl responses depending on the phase of the PMM, highlighting a climate-dependent modulation of ecosystem sensitivity to physical forcing.

**Table 5:** Summary of the GAMs (Gamma family, log-link function) applied hierarchically to disentangle the drivers of temporal Chl variations.

Model	Smooth	edf	Ref. df	F	*p*-value	*R*^2^ adj	%Dev exp	AIC	REML
A	Depth	4.02	4.97	164.6	< 2e-16	0.76	78.6	938.4	538
	MLD	3.87	3.98	221.4	< 2e-16				
	Ekman_Acu_	2.68	3.15	58.4	< 2e-16				
B	Depth	4.64	5.69	219.8	< 2e-16	0.80	84.2	718.4	405
	MLD	4.80	4.96	23.8	< 2e-16				
	Ekman_Acu_	4.85	4.96	80.3	< 2e-16				
	PMM	3.90	3.98	50.6	< 2e-16				
C	Depth	4.60	5.64	231.3	< 2e-16	0.80	84.3	713.4	397
	MLD	1.00	1.00	3.1	0.078				
	Ekman_Acu_*PMM	13.55	13.90	98.6	< 2e-16				

Estimated and reference degrees of freedom are denoted as edf and Ref.df, while percentage of deviance explained appears as %Dev exp. AIC denotes the Akaike Information Criterion, where lower values indicate a better trade-off between log-likelihood and model complexity. REML denotes the Residual Maximum Likelihood in which lower values indicate better relative fit.

## Discussion

Diverse types of governance converge in the Humboldt Archipelago to administer, in a sustainable fashion, the multiple ecosystem services it provides for human well-being, including marine reserves, benthic resource management areas (AMERB), and the recently declared multiple-use marine protected area (AMP-MU). For a successful coexistence of these diverse types of governance and the achievement of their main goal, the sustainability of the Humboldt Archipelago, a more comprehensive understanding of the ecosystem functioning is urgently needed. In particular, its basal trophic processes and coupling with environmental conditions, which vary over a wide range of vertical and temporal scales, ultimately sustain the biological complexity of the archipelago^24,33^. Presenting the most comprehensive oceanographic approach to date addressing the critical issue of spatial-temporal variations in marine productivity of the HA, this study indicated clear seasonal and interannual variations in phytoplankton biomass. Seasonal Chl dynamics were associated with the spring-summer shallowing of the MLD, while interannual Chl variations were associated with climate modulation exerted by the PMM on upwelling efficiency. However, the single sampling site might have underestimated the spatial complexity of Chl variations due to the topographically and bathymetrically heterogeneous archipelago, as confirmed by remote sensing (Figs. 3 and 4). In addition, nutrients’ information was not available, which would have supported interpretations about upwelling efficiency across temporal scales.

The spatial pattern of the correlation between CHL and the in situ Chl time series was strongest in the inner and northern section of the archipelago (Fig. 3) and suggests the occurrence of localized bio-oceanic effects affecting Chl concentration^21^. This may result from section-specific retention/dispersion mechanisms arising from the interaction between currents, upwelling filaments, and the highly irregular topography and bathymetry of the archipelago, which is strongly shaped by the submarine canyon located between the shoreline and Chañaral Island^18,21^. Indeed, while the submarine canyon may facilitate the intrusion of bottom water onto its eastern side and allow locally upwelled nutrient-rich water to enter from the south^18,21^, a relatively stable, likely oceanic, southeastward flow has been consistently observed to flow to the north of the island^21,24^. Whether by determining nutrient availability and water column stability, or retention/dispersion conditions, these oceanographic processes near Chañaral Island may foster a spatial mosaic of phytoplankton abundance and structure^12,34,35^. These processes might be particularly relevant in the southeast region which exhibited the highest negative bias (~-9 mg m^− 3^) during the satellite diagnosis. This was associated with the location of strong episodic Chl blooms in this southern subregion, indicative of changes in physical controls. Submesoscale structures originating from the interaction between surface currents and islands (Choros and Damas Islands in the current study) and leading to horizontal divergence might promote localized nutrients supply^36^. Alternatively, broader satellite images (Fig. 4) combined with the prevailing surface circulation pattern^21^, suggest an export of organic matter from the relatively more productive Coquimbo Bay system located at the south of the archipelago^20–22^. Further studies involving simultaneous genetic and isotopic characterization of phytoplankton and nutrients isotope signals might help elucidate the oceanographic teleconnection between the HA and the Coquimbo upwelling system.

We found strong photo-inhibition in the upper layer of the archipelago throughout the year (Fig. 5C, D,E), although the circadian cycle of phytoplankton cells might also lead to low fluorescence^37^. Although most empirical studies utilize phytoplankton biomass production or standing biomass as surrogate measures of productivity^38^, photoacclimation and shifts in phytoplankton community composition can partly decouple fluorescence and Chl estimates. Previous studies have reported that fluorescence quenching might substantially underestimate surface Chl concentration if not corrected^9,39–41^. In our study, the correction of fluorescence profiles was therefore essential to accurately resolve the temporal and vertical variability of phytoplankton biomass. The predominance of subsurface Chl maxima further suggests that phytoplankton growth is favored in deeper portions of the mixed layer where nutrient availability is higher and irradiance is reduced^42,43^. This pattern is consistent with the strong role of mixed-layer dynamics identified by the GAM analysis and supports the interpretation that seasonal shoaling and deepening of the MLD regulate the balance between nutrient supply, light exposure and biomass accumulation in the Humboldt Archipelago. During highly productive spring and summer periods, the potential additional input of allochthonous phytoplankton biomass exported from the southern Coquimbo Bay system may further contribute to sustaining the trophic functioning of this mesotrophic ecosystem.

Phytoplankton biomass was strongly seasonal in the archipelago with higher Chl values in spring and summer months, while also exhibiting interannual variations (Fig. 8). Furthermore, Chl concentration was maximal below the surface at intermediate depths and declined rapidly below this layer (Fig. 9). This suggests a vertically constrained productive layer, consistent with light availability and near-surface nutrient enrichment in the coastal upwelling system. According to the GAM results, 84.2% of the seasonal Chl fluctuations in the HA were associated with depth, MLD, and Ekman transport (Fig. 9). Access to nutrients retained from winter in deeper layers through vertical displacement of the MLD, was the driving mechanism, providing the physical background for optimum Chl periods in which solar radiation might act as an enabling factor^44^. Thus, during spring when sunlight is increasing but not maximal, the MLD is deep enough that phytoplankton can use relatively deep nutrients associated with the high salinity and low oxygen ESSW prevailing during winter^23,45^. During summer, the MLD is too shallow, and sunlight is at a maximum, leading to nutrient limitation at the end of the season. In winter, the MLD becomes too deep, enhancing access to nutrients, but the sunlight is minimal and Chl is low. This pattern indicates that phytoplankton biomass is favored under conditions of limited vertical mixing, whereas deeper MLDs are associated with reduced near-surface Chl concentrations^45^. Alternatively, nutrients might modulate the efficiency of phytoplankton utilization of sunlight as the MLD becomes shallower from spring to autumn^43^. The non-linear response of Chl to Ekman transport suggests that phytoplankton biomass is regulated not solely by the intensity of upwelling-favorable winds, but by upwelling efficiency^46^. According to these authors, whose study corresponds to an embayment shallower than the current sampling site, maximum Chl concentrations were related to the seasonal vertical migration of the MLD. In the current study, the smooth term for Ekman indicates that the optimal window for efficient access to nutrients and thereby for phytoplankton growth, occurs at intermediate transport values. In contrast, stronger winds may enhance offshore advection, deepen the mixed layer, or increase turbulent mixing, thereby reducing near-surface biomass accumulation as observed in other upwelling regions^47,48^. These results suggest the existence of a non-linear tipping point between positive (nutrient availability) and negative (biomass dilution) feedback of physical dynamics. Future studies should evaluate how residence time, turbulent mixing, light availability, and local retention processes interact to regulate this transition in coastal upwelling systems. In the Humboldt Archipelago, such thresholds are likely further modulated by the complex bathymetry and circulation associated with the submarine canyon and island system^19,22^, which may decouple nutrient supply from phytoplankton accumulation.

Besides revealing a strong nonlinear and non-additive effect on Chl (Table 5), the significant interaction between Ekman transport and the PMMi indicates that the Chl response to upwelling was highly dependent on the oceanic background state^28,30,32,49^. Moderate Ekman transport under positive PMM, and during La Niña and neutral El Niño conditions in years 2022 and 2024, resulted in the strongest positive effects on Chl, whereas intense upwelling under neutral or negative PMM conditions showed weak or even negative effects (Fig. 8E). These results suggest that favorable large-scale climatic conditions enhance the biological efficiency of upwelling through combined effects on nutrient supply, stratification, circulation and water residence time, while excessive mixing or unfavorable stratification might limit phytoplankton accumulation. Additional processes not explicitly resolved by the GAM framework can also contribute to interannual environmental variability. In particular, sub-to-mesoscale circulation and eddy kinetic energy can influence retention, offshore export and biomass aggregation within the HA, where local circulation is shaped by semi-permanent cyclonic eddy^19^. A recent study in the region showed that both Ekman transport and geostrophic transport exceeded their climatological means during the 2023 El Niño event^25^, supporting the notion that climate modes might alter both local upwelling intensity and mesoscale activity. In the California EBUS, the long-term trend of CHL has been linked to PMM modulation and its interaction with multiannual modes such as ENSO^32^. Consistent with this framework, our results indicate that for the study area and target periods, the PMM captures bio-oceanographic variability more efficiently than commonly used ENSO indices, although PMM and ENSO cannot be considered independent processes^50^. The mechanism underlying this climatic modulation remains unresolved. Previous studies have suggested that ENSO-PMM coupling might involve delayed atmospheric and oceanic responses affecting regional winds, SST and upper-ocean heat content. For example, Dewitte et al.^30^ proposed a lag phase of several months between ENSO and the PMM associated with poleward propagation of equatorially forced baroclinic waves, while Fan et al.^51^ suggested that the alignment of ENSO and PMM phases might amplify or damp the climate influence on regional forcing. Although the present dataset does not provide sufficient temporal degrees of freedom to explicitly resolve such lagged coupling mechanisms, our results support the hypothesis that low-frequency climate interactions might modulate the efficiency of local upwelling forcing and phytoplankton biomass accumulation in the Humboldt Archipelago.

## Conclusions

Phytoplankton biomass in the Humboldt Archipelago was strongly photoinhibited during daytime in the surface layer. On the temporal scale, phytoplankton biomass displays a strong seasonal pattern, with a maximum in spring associated with the vertical displacement of the mixed layer depth. Interannual variations indicated higher chlorophyll-a concentrations during La Niña and neutral El Niño periods. However, such interannual fluctuations must also consider the interaction between ENSO and the PMM, leading to changes in upwelling efficiency. The study demonstrates the significance of in situ time series for understanding the ocean response to climate modes.

## Methods

### Study area

In situ hydrographic measurements were conducted between August 2022 and December 2024, on the eastern side of Chañaral Island (− 29.04°S, − 71.54°W), the largest island (> 27 km^2^), and the northernmost marine protected area of the HA (Fig. 1; Table 1). A prolonged (two-year) La Niña event ended in December 2022, giving rise to neutral yet brief conditions. Subsequently, a coastal El Niño, prompted by PMM activity, developed off the northern coast of Peru in April 2023^28^, while a basin-scale El Niño was observed from June and lasted until December 2023^24^. Neutral El Niño conditions persisted during most of 2024.

### Satellite images

Climatologies of SST and sea surface chlorophyll (CHL) were derived from satellite observations to contextualize the hydrographic conditions observed during the study period in the archipelago and provide a broader understanding of the in situ sampling performed. The data were extracted from the GlobColour L3 product (https://data.marine.copernicus.eu/product/OCEANCOLOUR_GLO_BGC_L3_NRT_009_101), a daily, 300 m-resolution dataset from the OLCI A and B sensors. Due to the sparsity of daily data, the records were aggregated using monthly averaging. Data from August 2022 to December 2024 were used to match the in-situ sampling period. The data region was selected from − 71.82° to -71.29° for longitude and − 29.40° to -28.85° for latitude to provide a wider context around the sampling site at 29.04° S and 71.54° W.

### Atmospheric setting

The wind field was assessed using a meteorological station located on Chañaral Island, which started operations in October 2022 (Fig. 1B), two months after the onset of oceanographic measurements. To achieve a complete wind time series for Chañaral Island between August and December 2022, we used data from a second meteorological station located southward at Punta de Choros (29.2472°S; 71.4679°W), given the significant correlation observed between the two alongshore wind temporal series (r^2^ = 0.88; p-value < 0.001) (Sup. Mat. Figure 2). Due to the island’s north-south orientation, the alongshore wind was considered equivalent to the meridional component of the wind vector throughout the study.

The zonal (τ_x_) and meridional (τ_γ_) components of the wind stress were defined as follows:

1 $$\tau u={\rho _a}~{C_d}~u{U_{10}}~;~\tau v=~{\rho _a}~{C_d}~v{U_{10}}~~~$$

where ρa is air density (1.2 kg m^− 3^); C_d_ is a dimensionless drag coefficient; u and v are the zonal and meridional wind components, respectively; and U_10_ is the magnitude of the wind vector 10 m above sea level.

The drag coefficient C_d_ was calculated as a function of the wind velocity^52^, according to the following equations:

2 $${C_d}=0.29+\frac{{3.1}}{{{U_{10}}}}+\frac{{7.7}}{{U_{{10}}^{2}}} \times {10^{ - 3}},~for~{U_{10}}~ \leqslant ~6~m{s^{ - 1}}$$

3 $${C_d}=0.60+0.07{U_{10}} \times {10^{ - 3}},~for~6~m{s^{ - 1}} \leqslant {U_{10}}~ \leqslant ~26m{s^{ - 1}}$$

The surface Ekman transport components (*Ue, Ve*; m^2^ s^–1^) were calculated from the daily wind stress as:

4 $$~M~=~\frac{{~\tau ~}}{{{\rho _\omega }f}}~~~~~~~~~~$$

Where ρ$\:\omega\:$ is the seawater density (1025 kg m^− 3^), and *f* is the latitude-dependent Coriolis parameter, and varied as a function of the latitude. The depth of the surface Ekman layer, DE (units in meters), was calculated as in^53^ for latitudes (θ) outside ± 10° from the Equator:

5 $${D_E}=~\frac{{4.3}}{{\sqrt {sin~\left| \phi \right|~} }}~{U_{10}}$$

Water column mixing was estimated using the Brunt–Väisälä frequency, or buoyancy frequency. Solar irradiation data (Watt m^2^) were obtained from the meteorological station located on Chañaral Island from January 2023 to December 2024.

### Pacific Meridional Mode index

A PMM–like index (PMMi) was derived from local physical variables to represent the low-frequency atmospheric–oceanic state of coastal upwelling dynamics. Monthly means of MLD, surface wind speed, wind stress, Ekman transport, Ekman layer depth, and accumulated Ekman transport were first standardized (to zero mean and unit variance; z-score normalization) and combined using Principal Component Analysis (PCA). Monthly means of wind and derived variables were computed from daily wind data obtained from weather stations. Monthly MLD means were obtained from the Copernicus Marine Service merged product^54,55^, with a 1/4° resolution, surrounding the in situ sampling site (-29.033 to -29.117 S and − 71.633 to -71.528 W). PMMi > 0 represents intense southerlies, efficient Ekman transport, sustained accumulated Ekman transport, and elevated upwelling and nutrient availability. A negative PMMi (< 0) suggests the opposite of Niño-like conditions. The first principal component accounted for the largest fraction (66%) of the shared variance (Sup. Mat. Fig. S2), grouping the physical variables underlying the PMM effect on the ocean–atmosphere system^26^. It emphasizes the influence of regional forcing on basin-scale climate variability (i.e., ENSO) on coastal ecosystem processes^28,30,49^. Therefore, PC1 was retained as the PMMi.

### Hydrographic measurements

Hydrographic conditions were measured at a fixed sampling point (29.2472 °S; 71.4679 °W) near Chañaral Island using an RBRmaestro^3^: multi-channel logger (hereafter CTD) equipped with Coda (dissolved oxygen) and Turner Cyclops (fluorescence) sensors. The CTD provided vertical profiles (0–130 m) of temperature, salinity, dissolved oxygen, and fluorescence (Fig. 1; Table 1). CTD-fluorescence records were validated and converted to Chl using high-precision fluorometric measurements (see below).

Discrete oceanographic samples were collected at depths of 10, 30, 60, and 90 m with a Niskin bottle to provide dissolved oxygen and Chl samples (Table 1). The sampling depths for phytoplankton were designed to capture the vertical distribution in relation to the interaction between the depth of the photic layer and wind- and current-driven mixing. In the HCS, the depth of the photic layer has been reported to range between 40 and 100 m^56,57^. The concentration of Chl was determined from triplicate samples (100 mL) by filtering onto a GF/F filter (nominal pore size = 0.7 μm). Chl was extracted for 24 h in 90% acetone (v/v) and measured using a Turner fluorometer^58^. The accuracy of the Chl measurements was 0.17 mg Chl m^− 3^.

### Data analysis

Sea-surface chlorophyll data from remote sensing were used to evaluate the spatial representativeness of the sampling site within the broader region. Specifically, the time series of remotely sensed data at the grid point closest to the sampling site was compared with the remote sensing time series in each of the remaining grid cells. Two metrics were compared: the correlation (i.e., the Pearson product-moment correlation coefficient) between the two time series and the bias (i.e., the average difference between the two time series). These were both mapped spatially and averaged according to their distance from the sampling site.

To convert CTD-fluorescence records into Chl profiles, we used the methodology of^9^. It first establishes the instrumental offset (Offset, units mg m^− 3^), which is detected in the profile via the fluorescence value (Fluo) in deep waters (> 300 m). This is far below the photic layer, and thus, Chl concentration is considered negligible at these depths. Because the sampling site is at a depth of just 140 m, additional CTD casts were carried out on five occasions between December 2022 and December 2024 at deeper (450 m) stations located offshore of the study area (Fig. 1; Table 1). The offset was estimated as the average of CTD-fluorescence records between 300 and 400 m depth. CTD-casts were grouped by season, assigning the seasonal offset value (or the average) to those shallower CTD-casts without offset values, which were then subtracted from all profiles (Table 1). These corrected profiles were evaluated for fluorescence quenching^9^. Since quenching affects day profiles, night profiles obtained within 24 h are considered the reference. Seven day-night samplings were conducted over 24 h cycles, also considering the MLD to correct for the fluorescence quenching. The maximum fluorescence (Max_Fluo_) tends to define the deep quenching threshold, and thus, the depth of Max_Fluo_ (Z_MaxFluo_) is also considered in the fluorescence quenching correction (Table 2). For this calculation, the MLD was defined as:

6 $$MLD=Temperatur{e_{0 - 10m}} - 0.5^\circ C$$

Then, the MLD is divided into two layers: the upper layer between the surface and Z_MaxFluo,_ and the lower layer between Z_MaxFluo_ and MLD. The ratio between day and night fluorescence profiles is calculated and arranged into upper or lower nominal layers of the MLD (Table 2). Once Max_Fluo_ is identified, this value is extrapolated upward to correct for quenching effects on day profiles. Only profiles in which Z_MaxFluo_ was shallower than MLD were corrected for fluorescence quenching with the assumption that Chl is homogeneously distributed in the upper layer^9^. A linear regression was thereafter performed between quenching-corrected fluorescence profiles and discrete fluorometric measurements of Chl. Linear regressions were performed for each survey, yielding slopes ranging from 0.41 to 2.9 (Table 1). Correlation (adj) coefficients of linear regressions between fluorescence casts and discrete Chl measurements varied between 0.75 and 0.99 (Sup. Mat. Table S1), indicating a relatively high performance of the fluorescence CTD sensor.

Chl profiles were displayed in vertical sections to evaluate temporal and vertical distributions over the assessed period. Besides seasonal transitions, this period also encompassed different years, ENSO, and PMM phases that may influence the oceanic background for seasonal dynamics in the HA. Since years and ENSO are correlated (i.e., each year has a specific ENSO phase), we used year and season as categorical factors. Phytoplankton biomass responds to both the concurrent ocean state and environmental history, which determines local physical conditions (depth, temperature, salinity, MLD, wind speed, wind stress, Ekman transport, Ekman layer, and solar radiation), as well as low-frequency local climatic and remote forcing over seasonal and interannual time scales. To quantify these fluctuations and disentangle the relative roles of local versus remote forcing, Generalized Additive Models (GAM) were fitted following a temporal hierarchical approach (Table 3). Thus, to evaluate the effect of the concurrent ocean state, the baseline physical model (Model A) included depth, MLD, and Ekman transport accumulated over the previous five days. The effects of temperature and salinity on the vertical physical structure are captured by the MLD and included in the model. A subsequent Model B evaluated the role of PMMi as a low-frequency climatic modulator of local forcing. A third and final Model C explicitly represented the interaction between local upwelling dynamics and the climatic state through a tensor product smooth between Ekman transport and PMMi. It should be noted that although hydrographic measurements were collected in 2022 exclusively during the winter and spring seasons (Table 1), GAM-adjusted predictions were generated for all possible combinations of season and year during the creation of the prediction grid. Solar irradiance was not included in the GAM analysis because data were unavailable in 2022. To preserve interannual comparability, irradiance effects were instead explored separately using the 2023–2024 data subset and discussed as a potential mechanistic driver of Chl variability. Furthermore, since oxygen is not a direct driver of Chl but, in part, a product of it, oxygen was not included in the analysis. Basis dimensions (k) were initially defined conservatively to avoid overfitting and subsequently evaluated using diagnostic R routines (mgcv package (gam.check)). When effective degrees of freedom (edf) approached the maximum basis dimension (k′) together with low k-index values, k values were slightly increased to improve model flexibility and better capture non-linear responses. Final model selection was based on REML, AIC, explained deviance, and residual diagnostics (Table 5). As part of the GAM process, concurvity tests were generated to detect autocorrelation between continuous descriptors (Sup. Mat. Table S2).

## Supplementary Information

Below is the link to the electronic supplementary material.

Supplementary Material 1

## Data Availability

The data sets supporting this study include MLD data from the Copernicus Marine Data Store (Guinehut et al., 2012; Mulet et al., 2012). Climatologies of sea surface chlorophyll were obtained from NASA satellite observations (Kilpatrick et al., 2015, 2019; NASA Ocean Biology Processing Group, 2022). The study also incorporated in situ data of oceanographic and biological variables (Aguilera et al., 2025 and 2025a). Data and metadata are available via Zenodo at https://doi.org/10.5281/zenodo.19099588.
