# Supplementary material for: Climate-modulated upwelling drives phytoplankton variability and biomass connectivity in the Humboldt Archipelago coastal system
Source: Sci Rep. 2026 Jun 11;16:26562. doi: 10.1038/s41598-026-56707-y (PMC13503702; doi:10.1038/s41598-026-56707-y)
Supplement: Supplementary file 1 [file 41598_2026_56707_MOESM1_ESM.docx]

Supplemntary material:

Climate-modulated upwelling drives phytoplankton variability and biomass connectivity in the Humboldt Archipelago coastal system

**Figure S1:** Vertical distribution of the Max_Fluo_ within the upper layer in relation to the upper and lower nominal layers of the mixed layer. Both the upper and lower layers show day-night fluorescence ratio values < 1, and Max_Fluo_ tends to be found in the lower nominal layer of the mixed layer.

**Figure S2:** PCA of monthly means of the atmospheric-oceanic variables from August 2022 to December 2024. PC1 retained the largest proportion of variance and was used as the PMMi.

**Table S1:** Summary of statistical results of the linear regressions between corrected fluorescence and fluorometric Chl measurements (bold). For dates without fluorometric Chl measurements, the regression slope was assigned using either hierarchical cluster matching or seasonal records.

| Sampling date | Regression | | | Slope |
| --- | --- | --- | --- | --- |
|  | Adj R^2^ | Slope | n |  |
| 2022-08-03 |  |  |  | 0.64 |
| 2022-09-02 |  |  |  | 2.50 |
| 2022-11-25 | 0.75 | 0.68 | 18 | **0.68** |
| 2022-12-16 |  |  |  | 0.66 |
| 2023-04-02 | 0.96 | 0.83 | 24 | **0.83** |
| 2023-05-10 | 0.99 | 0.64 | 18 | **0.64** |
| 2023-06-20 | 0.95 | 0.67 | 18 | **0.67** |
| 2023-07-14 | 0.96 | 0.66 | 24 | **0.66** |
| 2023-10-13 |  |  |  | 0.67 |
| 2023-11-30 | 0.97 | 2.90 | 24 | **2.90** |
| 2024-01-09 | 0.99 | 2.50 | 18 | **2.50** |
| 2024-02-05 | 0.99 | 1.23 | 18 | **1.23** |
| 2024-03-07 | 0.77 | 1.36 | 18 | **1.36** |
| 2024-04-02 | 0.85 | 0.90 | 18 | **0.90** |
| 2024-05-12 |  |  |  | 2.50 |
| 2024-06-21 |  |  |  | 1.36 |
| 2024-07-14 |  |  |  | 0.66 |
| 2024-08-29 |  |  |  | 0.90 |
| 2024-10-06 |  |  |  | 0.90 |
| 2024-11-08 |  |  |  | 0.64 |
| 2024-12-04 |  |  |  | 2.90 |

**Table S2:** Results of concurvity test on hierarchical GAM conducted to evaluate temporal variations of Chl in the Humboldt Archipelago. Concurvity values are categorized as optimum (0 to 0.5), moderate (0.5 to 0.8), high (0.8 to 0.9), or severe (> 0.9). High concurvity values likely reflect physically coupled environmental processes rather than purely statistical redundancy, particularly because PMM modulates the mean state of local wind forcing, mixed-layer structure, and Ekman transport.

| Model | Smooth term | Estimated concurvity |
| --- | --- | --- |
| B | s(Depth) | <0.001 |
| B | s(MLD) | 0.75 |
| B | s(EkmanAcu) | 0.98 |
| B | s(PMM) | 0.90 |
| C | s(Depth) | 0.05 |
| C | s(MLD) | 1.00 |
| C | te(EkmanAcu, PMM) | 0.59 |
